# Suture of Nerves

**Published:** 1886-07

**Authors:** 


### Selections.

### Suture of Nerves.

Koppenschaar has made numerous experiments proving that complete sensation will follow the suture of nerves. Five cases have been operated on by Iterson, of Leyden, with successful results.

*Case 1.*—Median nerve destroyed above the radio-carpal articulation. Suture operation eight weeks after. Proximal end of nerve was found enlarged, the distal end flattened. The free portions were trimmed off and sutured. In three months, the sensation was much improved, but never became normal.

*Case 2.*—Radial nerve destroyed in middle of forearm. Thirty-two days after, the nerve was sutured. In fifty days, sensation returned. By repeated Faradization, motion was also restored.

*Case 3.*—Destruction of median nerve in lower third of forearm. Fourteen weeks after, the nerve was united by sutures. In two days, sensation returned. In two weeks, sensation was again partly lost in the middle and ring fingers. Motion returned, and gradually improved with exercise and Faradism.

*Case 4.*—Destruction of median nerve. Nerve united. Sensation has not yet returned after a lapse of four months.

*Case 5.*—Destruction of median nerve in same region. Six weeks after, the nerve was united by sutures. After the expiration of four weeks, sensation became normal.

In regard to the histological development of the new nerve formation, Koppenschaar states that there is a granular deposit not only in the protoplasm, but also in the medullary substance. The nuclei in the medullary substance were surrounded by small masses of protoplasm. It was not possible to determine whence the nuclei came. He also considers it impossible for nerves to unite by first intention, although it is difficult to explain certain cases (case 3) otherwise.

In cases where there has been an extensive loss of substance, he advises the method of Vanlair, who surrounds the injured nerve at the place of suture by a piece of decalcined bone.—*Gazz. Med. di Roma.*

